# Anatomically Based Ablation of Left Ventricular Summit Premature Ventricular Complexes Guided by Intracardiac Echocardiography

**DOI:** 10.19102/icrm.2024.15024

**Published:** 2024-02-15

**Authors:** Nikhil Sharma, Kristie M. Coleman, Gregory Cunn, Jeremy Kleiman, Stavros E. Mountantonakis

**Affiliations:** 1 Department of Electrophysiology, Lenox Hill Hospital, Northwell Health, New York, NY, USA

**Keywords:** Intracardiac echocardiography, left ventricular summit, non-invasive electroanatomical mapping

## Abstract

Catheter ablation of premature ventricular contractions (PVCs) arising from the left ventricular summit (LVS) presents technical challenges due to the regional anatomy and frequent intramural site of origin (SOO). Intracardiac echocardiography (ICE) and the CARTOSOUND^®^ (Biosense Webster, Diamond Bar, CA, USA) module allow the operator to directly reconstruct and visualize the dimensions and orientation of the LVS live and present it in relation to neighboring structures. We retrospectively reviewed consecutive cases between January 2021 and December 2022 of patients undergoing PVC ablation for a presumed LVS origin. The LVS was reconstructed by creating a three-dimensional representation of the left ventricular septum, using two-dimensional ICE sections. The earliest site in each chamber was tagged on the reconstructed LVS, and the presumed SOO was localized using a geometrical center point from all sites. Ablation was first delivered to the earliest site, except when the presence of coronary branches precluded radiofrequency delivery within the great cardiac vein. Of 20 patients (8 women, 62.4 ± 7.1 years old) with a presumed LVS origin, 12 had PVC recurrence within the monitoring period after the initial ablation for 192.5 ± 37.2 s at the earliest site. Among them, earliest activation was seen at the sinus of Valsalva (SoV), coronary venous system (CVS), and left ventricular endocardium (LVE) in four, six, and two patients, respectively. Using the reconstructed LVS, the anatomically closest site to the SOO was identified in the SoV, CVS, and LVE in four, two, and six cases, respectively. Throughout the study period (14.5 months; range, 9.3–19.7 months), 17 patients (85%) had complete elimination of PVCs as evaluated by 24-h event monitors at the 12-month visit. In 50% of cases, among patients in whom ablation at the earliest signal was unsuccessful, the site of successful ablation did not correlate with the second earliest signal or had no identifiable signal during initial activation mapping. The reconstructed LVS not only guided activation mapping but also identified sites proximal to the center point that had either a late activation signal, a low-amplitude signal, or no signal at all.

## Introduction

**Figure 1:**
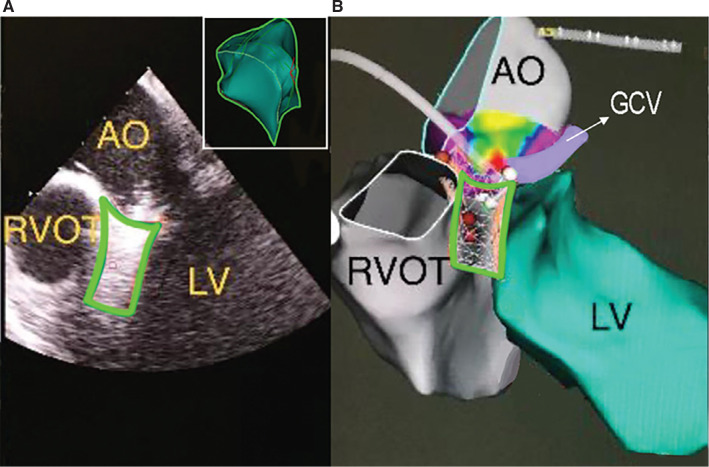
**A:** Intracardiac echo image of the left ventricular summit (LVS) and its anatomic relationship to surrounding structures. Inset showing a three-dimensional reconstruction of the LVS. **B:** CARTOSOUND^®^ image of the LVS and surrounding structures. *Abbreviations:* AO, aorta; GCV, great cardiac vein; LV, left ventricle; RVOT, right ventricular outflow tract.

Catheter ablation of premature ventricular contractions (PVCs) arising from the left ventricular summit (LVS) presents technical challenges due to the regional anatomy and frequent intramural site of origin (SOO).^1^ Various ablative strategies have been employed, including ablation from neighboring structures such as the sinus of Valsalva (SoV), right ventricular outflow tract (RVOT), or coronary venous system (CVS); ethanol infusion through septal venous perforators; and radiofrequency (RF) delivery through a guidewire inserted into small branches of the coronary sinus system.^2^ A clear understanding of the anatomy and patient-specific variations is essential to form an effective ablation strategy, which is often anatomical, targeting sites with the closest proximity to the presumed SOO. Nevertheless, the three-dimensional (3D) anatomy of the LVS is not imaged independently with endocardial mapping but rather deducted from mapping of the surrounding chambers—namely, the RVOT, left ventricular endocardium (LVE), and SoV **(Figure 1)**.

Intracardiac echocardiography (ICE) and the CARTOSOUND^®^ (Biosense Webster, Diamond Bar, CA, USA) module can allow the operator to directly reconstruct and visualize the dimensions and orientation of the LVS live and present it in relation to neighboring structures. We examined the utility of 3D reconstruction of the LVS using CARTOSOUND^®^ to identify sites with the closest anatomical proximity to the presumed SOO.

## Methods

We retrospectively reviewed consecutive cases between January 2021 and December 2022 of patients undergoing PVC ablation for a presumed LVS origin. The LVS origin was assumed when complete activation mapping of the RVOT, SoV, CVS, and LVE revealed at least three sites with a minimum activation of –25 ms pre-QRS and an activation difference between sites of <5 ms. The study protocol received exempt approval from the Northwell Health Institutional Review Board; an informed consent waiver was received as the study was deemed to be of minimal risk to participants.

The LVS was reconstructed by creating a 3D representation of the left ventricular septum, using two-dimensional ICE sections obtained from three different locations—namely, (1) the high right atrium/superior vena cava junction, (2) the right ventricular inflow, and (3) the RVOT.^3^ The earliest site in each chamber was tagged on the reconstructed LVS, and the presumed SOO was localized using a geometrical center point from all sites. Ablation was first delivered to the earliest site, except when the presence of coronary branches precluded RF delivery within the great cardiac vein. In cases without successful elimination or recurrence, lesions were then delivered to the site anatomically nearest to the SOO, irrespective of activation at that site. The duration and number of lesions were left to the operator’s discretion but included at minimum a 1-min lesion starting at 30 W and achieving a ≥15-Ω impedance drop.

Acute procedural success was defined as non-inducibility of the clinical PVC after a 30-min waiting period. Twenty four–hour event monitoring was performed at 6- and 12-month follow-up visits.

## Results

Of 20 patients (8 women, 62.4 ± 7.1 years old) with a presumed LVS origin, 12 had PVC recurrence within the monitoring period after initial ablation lesions were delivered for 192.5 ± 37.2 s at the earliest site. Of those, earliest activation was seen at the SoV, CVS, and LVE in four, six, and two patients, respectively. Using the reconstructed LVS, the anatomically closest site to the SOO was identified in the SoV, CVS, and LVE in four, two, and six cases, respectively. The site anatomically closest to the SOO correlated with the second earliest activation in the same chamber in 7 out of 12 cases (58.3%). Particularly in the cases where SoV was identified as a closest site by using the reconstructed LVS, no signal was identified during initial activation mapping in two out of four (50%) cases. Similarly, successful ablation at the LVE site was not associated with an early signal in four out of six (66.6%) cases. Ablation in the anatomically proximal site for a mean of 443.4 ± 208.1 s resulted in the abolition of the PVCs in 9 out of 12 cases (75%). There were no known acute or long-term complications recorded in these cases.

All anti-arrhythmic drugs were discontinued. Throughout the study period (14.5 months; range, 9.3–19.7 months), 17 patients (85%) had complete elimination of PVCs as evaluated by 24-h event monitors at the 12-month visit.

## Discussion

Ablation of LVS PVCs, especially those arising from the septal aspect, is technically challenging due to the proximity of the coronary arteries and epicardial fat. A deep intraseptal origin often precludes effective lesion delivery to successfully eliminate these PVCs.^4^ Ablative strategies focus on delivering lesions that can penetrate to the true SOO using activation mapping as a starting point. Nevertheless, activation mapping is often challenging due to poor sampling and/or due to the presence of small, multicomponent, or far-field signals, especially in the SoV area, that often go unnoticed.^5^ Additionally, conduction within the septum may not always be circumferential due to preferential conduction, and arrhythmia breakout from an intramural origin may not always correlate with the site closest to the SOO.^2^ The anatomy of the outflow tracts often precludes complete electroanatomic mapping, and, in the absence of a signal, critical sites for ablation are not identified. Our study demonstrates how ICE-guided mapping combined with CARTOSOUND^®^ allows the operator to image the full geometry of the LVS and localize its center point. The reconstructed LVS not only guided activation mapping but also identified sites proximal to the center point that had either a late activation signal, a low-amplitude signal, or no signal at all. In 50% of cases, among patients in whom ablation at the earliest signal was unsuccessful, the site of successful ablation did not correlate with the second earliest signal or had no identifiable signal during initial activation mapping.

### Limitations

We present our technique for localizing the geometrical center point of the LVS based on the regional anatomy to identify proximal ablation targets as a proof of concept. The results of this study are based on a small retrospective series, and therefore a larger study needs to be conducted to determine the applicability of this technique for ablating LVS PVCs. Four-dimensional intracardiac echo will provide the operator with a more accurate representation of LVS and the ability to quantify the center point.
